# Intraocular Delivery of a Collagen Mimetic Peptide Repairs Retinal Ganglion Cell Axons in Chronic and Acute Injury Models

**DOI:** 10.3390/ijms23062911

**Published:** 2022-03-08

**Authors:** Marcio Ribeiro, Nolan R. McGrady, Robert O. Baratta, Brian J. Del Buono, Eric Schlumpf, David J. Calkins

**Affiliations:** 1 Department of Ophthalmology and Visual Sciences, Vanderbilt Eye Institute, Vanderbilt University Medical Center, AA7103 MCN/VUIIS, 1161 21st Ave. S., Nashville, TN 37232, USA; marcio.ribeiro@vumc.org (M.R.); nolan.mcgrady@vumc.org (N.R.M.); 2 Stuart Therapeutics, Inc., 411 SE Osceola St., Suite 203, Stuart, FL 34994, USA; bob@stuarttherapeutics.com (R.O.B.); brian@stuarttherapeutics.com (B.J.D.B.); eric@stuarttherapeutics.com (E.S.)

**Keywords:** neuroprotection, collagen mimetic peptides, glaucoma, optic neuropathy, collagen reparative, extracellular matrix, optic nerve crush

## Abstract

Vision loss through the degeneration of retinal ganglion cell (RGC) axons occurs in both chronic and acute conditions that target the optic nerve. These include glaucoma, in which sensitivity to intraocular pressure (IOP) causes early RGC axonal dysfunction, and optic nerve trauma, which causes rapid axon degeneration from the site of injury. In each case, degeneration is irreversible, necessitating new therapeutics that protect, repair, and regenerate RGC axons. Recently, we demonstrated the reparative capacity of using collagen mimetic peptides (CMPs) to heal fragmented collagen in the neuronal extracellular milieu. This was an important step in the development of neuronal-based therapies since neurodegeneration involves matrix metalloproteinase (MMP)-mediated remodeling of the collagen-rich environment in which neurons and their axons exist. We found that intraocular delivery of a CMP comprising single-strand fractions of triple helix human type I collagen prevented early RGC axon dysfunction in an inducible glaucoma model. Additionally, CMPs also promoted neurite outgrowth from dorsal root ganglia, challenged in vitro by partial digestion of collagen. Here, we compared the ability of a CMP sequence to protect RGC axons in both inducible glaucoma and optic nerve crush. A three-week +40% elevation in IOP caused a 67% degradation in anterograde transport to the superior colliculus, the primary retinal projection target in rodents. We found that a single intravitreal injection of CMP during the period of IOP elevation significantly reduced this degradation. The same CMP delivered shortly after optic nerve crush promoted significant axonal recovery during the two-week period following injury. Together, these findings support a novel protective and reparative role for the use of CMPs in both chronic and acute conditions affecting the survival of RGC axons in the optic projection to the brain.

## 1. Introduction

The extracellular matrix (ECM) is a biochemically active scaffold composed of proteins and polysaccharides critical for maintaining both biophysical stability and the availability of intercellular signals [1,2]. In both chronic and acute conditions of the central nervous system (CNS), biochemical degradation and remodeling of the ECM are important factors in promoting local inflammation and other pathogenic elements that impede neurite and synapse survival, axonal repair, and regeneration of circuits and projections [3,4,5]. Thus, new approaches that bolster matrix integrity and function could also promote the repair and survival of both individual neurons and the larger networks they form [2,6,7].

Collagen is the most abundant of the fibrous proteins of the ECM, which also include fibronectin and vitronectin. Collagen’s characteristic triple helical structure, present in all collagen domains, includes a set of three polypeptide chains comprising repeating sequences of glycine-x-y triplets, in which x and y generally represent proline and hydroxyproline [8]. Damage to ECM collagen is associated with age-related increases in susceptibility to injury or disease and slows the recovery of tissues to homeostatic balance [9,10]. By specifically annealing to and repairing fragmented triple helical collagen, collagen mimetic peptides (CMPs) show great promise as therapeutics that counter ECM degradation [11,12]. Single-strand CMPs incorporating fractions of type I collagen intercalate into fragmented triple helices to restore native structure and function [13,14,15], which is an important step given type I collagen’s ubiquitous expression, bioavailability, and low antigenicity. Reformation of helical collagen could also promote homeostatic intercellular signaling and reduce ligand binding sites for inflammatory mediators in local tissues [16]. 

As in other regions of the CNS, the ECM of the retina and optic nerve is also abundant in collagen, containing highly concentrated regions of collagen types I, III, IV, V, and VI [17,18]. In recent work, we found that ocular delivery of a CMP of type I collagen prevented degradation of axonal anterograde transport from retina to central brain targets along the optic nerve following a brief period of induced elevation in intraocular pressure (IOP) [19], using the widely prevalent microbead model of experimental glaucoma [20,21]. This is an important finding for several reasons. Degradation of anterograde transport is one of the earliest signs of functional axonopathy in glaucoma and is an antecedent to frank nerve degeneration and tissue loss; preventing transport degradation abates subsequent stages [22,23,24,25,26,27]. Additionally, sensitivity to IOP is a hallmark feature of glaucomatous optic neuropathy (or glaucoma), the world’s most prevalent cause of irreversible blindness [28]. Thus, current treatments seek to lower IOP through topical pharmaceuticals, surgery, or both. Even so, an estimated 40–50% of those with the disease will progress to irreversible vision loss [29,30]. In glaucoma, stress due to IOP sensitivity is conveyed to RGC axons at the optic nerve head, where the extra-axonal milieu contains a concentrated density of collagen secreted from a lateral plexus of astrocyte glial cells [31,32]. Glaucoma disrupts this collagen as part of the protease-mediated breakdown and deposition of the new ECM [33,34,35,36,37]. Finally, elevated IOP increases collagens I, IV, and VI in the retina and optic nerve head as part of a broader program of ECM remodeling [31,36,38]. Similarly, in traumatic optic neuropathy, approximately 50% of patients lose vision immediately due to acute loss of RGC axons, and 10% of patients exhibit delayed vision loss with optic nerve pathology weeks later [39]. Disruptions in ECM collagen and the ensuing breakdown of the ECM occur more rapidly in ocular trauma, exacerbating stress to RGC axons and accelerating degeneration [7]. 

The optic projection as part of the CNS does not regenerate intrinsically [40,41], necessitating new treatments that promote the survival or repair of RGCs and their axons in congenital, chronic, or traumatic conditions [40,42]. Given the importance of identifying new therapies, here we test the capacity of another novel CMP of collagen (CMP 13A) to promote RGC axonal repair based on the promising results of our recent study using our first CMP (CMP 03A) [19]. As before, we find that ocular delivery of a CMP via intravitreal injection prevents degradation of anterograde transport following three weeks of elevated IOP. However, we also report that the same CMP promotes significant axonal outgrowth following acute optic nerve crush. Thus, CMPs may have broad use as neuro-reparative agents in both chronic and acute conditions that challenge the survival of RGCs and their axons in the optic projection to the brain.

## 2. Results

### 2.1. Collagen Mimetic Peptide Improves Axon Transport in Experimental Glaucoma

**Figure 1:**
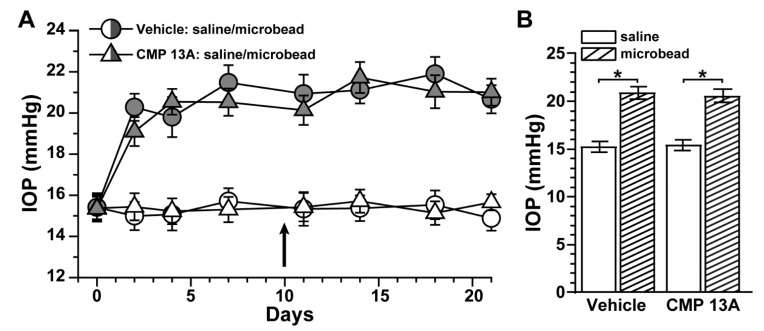
Collagen mimetic peptide does not influence IOP. (**A**) Mean daily intraocular pressure (IOP) in mice treated with vehicle (○, n = 6) vs. CMP 13A (Δ, n = 7) prior to (day 0) and following (days ≥ 1) a single unilateral injection of either polystyrene microbeads or an equivalent volume of saline in the contralateral eye; intravitreal injection of CMP or vehicle occurred on day 10. (**B**) Microbead injection significantly elevated IOP for both cohorts: vehicle (20.88 ± 0.67 vs. 15.27 ± 0.59 mmHg; *, *p* < 0.001); CMP 13A (20.58 ± 0.68 vs. 15.41 ± 10.57 mm Hg; *, *p* < 0.001). IOP did not differ between cohorts for either the saline- or microbead-injected eye (*p* ≥ 0.33). Data = mean ± SEM.

Active anterograde axonal transport from retina to central brain targets degrades early in RGC degeneration during experimental glaucoma [22,23,40,43]. Recently, we found that ocular delivery of a CMP (CMP 03A, (Pro-Pro-Gly)_7_, 100 µM) prevented the degradation of RGC axonal anterograde transport following three weeks of elevated intraocular pressure (IOP) induced by microbead occlusion [19]. In that same study, we also found that additional CMP sequences promote neurite outgrowth from dorsal root ganglia explants under conditions of MMP-1-induced collagen degradation. Here, we tested whether one of these CMPs (CMP 13A, (cis-Flp-Hyp-Gly)_7,_ 100 µM) also protects RGC axonal transport following elevations in IOP using microbead occlusion as described in mice [20,21,44]. Prior to the experiment (day 0), baseline IOP for each eye did not differ between vehicle and CMP cohorts (*p* ≤ 0.95; Figure 1A). Microbead injection induced a significant 34–37% elevation in IOP compared to the contralateral saline-injected eye that persisted through the 3-week experimental period (*p* < 0.001; Figure 1A). For both the microbead and saline eyes, IOP did not differ between vehicle and CMP cohorts (*p* ≥ 0.33); intravitreal injection of either vehicle or CMP 13A (day 10) had no measurable influence on IOP for either eye.

**Figure 2:**
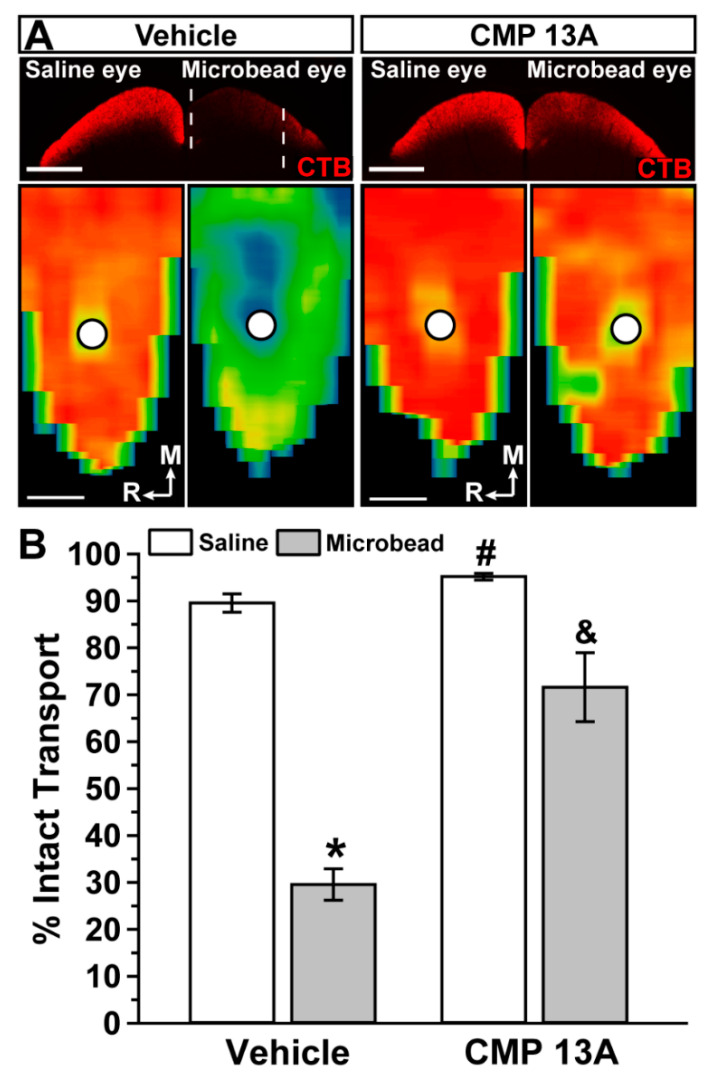
Collagen mimetic peptide protects anterograde axon transport. (**A**). Single sections through superior colliculus (top) showing regions of intact CTB transport (red) for mice receiving either vehicle (n = 6) or CMP 13A (n = 7) via intravitreal injection. In vehicle mice, the colliculus serving the optic projection from the saline-injected eye had fully intact transport, while microbead-induced IOP elevation in the fellow eye created regions of degraded transport (dashed lines). Retinotopic maps (bottom) reconstructed from serial sections through colliculus show levels of intact CTB signal ranging from 0% (blue) to 50% (green) to 100% (red). Medial (M) and rostral (R) orientations are indicated, as is the representation of the optic disc (white circles). Scale = 500 μm. (**B**). For vehicle-treated mice, intact transport declined by 67% with microbead-induced elevations in IOP compared to colliculus from the saline eye (*, *p* < 0.0001), while microbead eyes from the CMP 13A cohort demonstrated a two-fold improvement in transport compared to vehicle microbead (&, *p* = 0.002) and did not differ significantly from intact transport in the vehicle saline eye (*p* = 0.06). Interestingly, transport from the saline eye in the CMP 13A cohort exceeded that from the saline eye in the vehicle cohort (#, *p* = 0.01). Data = mean ± SEM.

Transport deficits to the superior colliculus occur within 2–3 weeks of microbead-induced IOP elevations in mice; actual axon degeneration in the optic nerve follows weeks later [43,45,46]. Pharmaceuticals that stop transport degradation also prevent subsequent axon and somatic degeneration [26,27,47,48,49]. As expected from our recent similar study [19], in mice with intravitreal injection of vehicle, three weeks of microbead-induced IOP elevation reduced RGC axonal transport from the retina to the colliculus, causing gaps in the retinotopic representation of intact CTB transport (Figure 2A). Intravitreal injection of CMP 13A appeared to reduce degradation, with the retinotopic map of intact transport from the microbead eye similar to the saline eye. When quantified, transport from vehicle-treated eyes with elevated IOP diminished by 67% (*p* < 0.0001, Figure 2B), consistent with our recent published results [19,43,45]. Compared to vehicle, intravitreal injection of CMP 13A significantly improved transport from microbead eyes by two-fold (*p* = 0.002), though intact transport did not reach saline-eye levels (*p* = 0.04). Interestingly, transport from the saline eye was also significantly greater than in the vehicle cohort (*p* = 0.01). 

### 2.2. Collagen Mimetic Peptide Repairs Axons following Optic Nerve Crush

**Figure 3:**
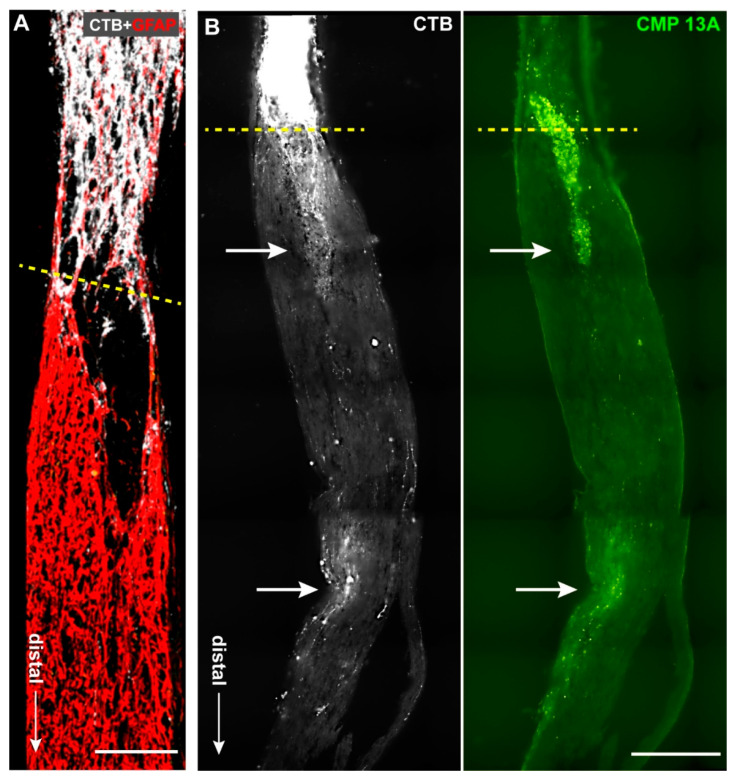
Collagen mimetic peptide promotes axon repair following nerve crush. (**A**) Stitched montage of confocal micrographs of longitudinal section through optic nerve two weeks following crush from eye receiving an intravitreal injection of vehicle (DMSO) three days after the injury. Axons containing cholera toxin B (CTB, false color white) extend to the site of crush (dashed line) but generally not beyond distally towards the brain (arrow). Astrocyte glia labeled for glial fibrillary acidic protein (GFAP, red) are shown for comparison. (**B**) In contrast, CTB-containing axons in section of nerve from eye receiving CMP 13A extend beyond the crush site and are apparent even at more distal locations along the nerve. Repaired axons are largely coincident with localized patches of CMP 13A (green), visualized through its attached chromophore (Tide Fluor™ 2, see Methods). Scale = 200 μm (**A**) or 100 μm (**B**).

Optic nerve crush (ONC) is a useful model of traumatic optic neuropathy and for assessing reparative and regenerative strategies [50]. Axon degeneration in this injury model also involves degradation of matrix collagen associated with robust tissue remodeling [7,47,51]. Two weeks following retrobulbar nerve crush, sections through nerves from eyes receiving intravitreal injection of vehicle (three days after crush) demonstrated very limited extension of CTB-labeled axons beyond the crush site (Figure 3A), as expected at this time in this model [50]. In contrast, nerves from eyes receiving CMP 13A (200 µM) showed a much higher density of axons extending distally beyond the crush towards central brain targets (Figure 3B). Interestingly, extending axons tended to colocalize with CMP 13A itself, which was apparent in small, concentrated patches as far as 0.5 mm beyond the crush site. We quantified the degree of colocalization by measuring (in arbitrary units) the intensity of CMP 13A puncta in CTB+ axons and averaging this factor for each nerve section. When compared to the length of each CTB+ axon colocalizing with CMP 13A, the resulting regression was not significant (*p* = 0.18, data not shown). Below, we discuss possible interpretations of this trend. 

**Figure 4:**
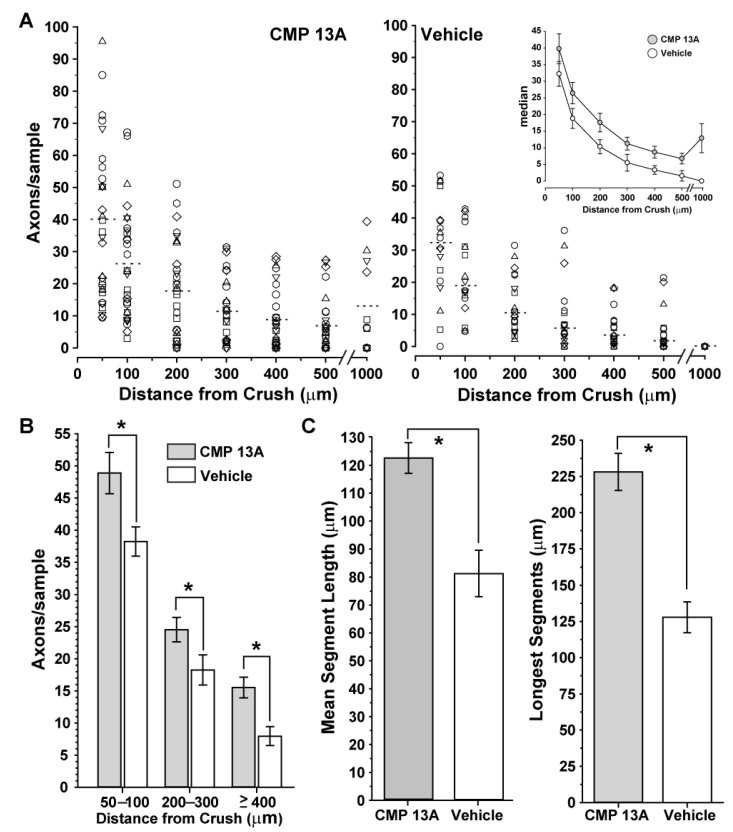
Collagen mimetic peptide increases axon recovery following nerve crush. (**A**) Number of CTB+ axon segments at specific distances distal to the injury site two weeks following optic nerve crush in individual samples (symbols), normalized to nerve width as described (see Methods). At each location, the median number of scored axons (dashed lines) in nerves from CMP 13A eyes (left) exceeded that in nerves from DMSO eyes (right); this trend is summarized in the inset. (**B**) The number of CTB+ axon segments above the median (top 50%) from (**A**) averaged at distances from crush site as indicated. Nerves from eyes receiving CMP 13A demonstrate a significantly greater number of axons at each location (*, *p* ≤ 0.02). (**C**) CTB-containing RGC axon segments in nerves from eyes receiving intravitreal CMP 13A extend further compared to axon segments from vehicle-treated eyes, both by mean length and the longest 25 segments in each group (*, *p* < 0.001).

Next, we quantified the number of CTB+ RGC axon segments at discrete distances distal from the crush site (towards the brain). Nerves from eyes receiving CMP 13A demonstrated more axon segments than nerves from vehicle eyes at each location examined, with some samples having CTB+ segments as far as 1 mm distal from the injury (Figure 4A). Even so, in both cohorts, there were a number of samples with few, if any, axons distal from the crush site, leading to substantial variability. To better estimate the magnitude of the effect due to CMP 13A compared to vehicle, we pooled the measurements above the median at each location into three bins: 50–100 µm, 200–300 µm, and 400 µm and further from the injury site. For these locations, nerves from CMP 13A-treated eyes demonstrated a 29%, 39%, and 96% greater abundance of CTB-containing axon segments, respectively (Figure 4B, *p* ≤ 0.02). Intact axon segments in these nerves were also longer than those in nerves from vehicle-treated eyes. The mean length of intact axon segments in nerves from CMP 13A-treated eyes was 51% greater than that in the vehicle cohort: 122.6 ± 5.5 vs. 81.1 ± 8.3 µm (*p* < 0.001; Figure 4C, left). Similarly, the mean for the longest 25 segments in the CMP 13A nerves was nearly 80% greater than that for vehicle nerves: 228.1 ± 12.9 vs. 127.8 ± 10.5 µm (*p* < 0.001; Figure 4C, right).

## 3. Discussion

Optic neuropathies are a major cause of vision loss and disability worldwide. The retinal ganglion cell (RGC) axon is central to pathogenesis in optic neuropathy; degradation of axon function leads to frank axon degeneration, so that preserving axon physiology slows subsequent stages [22]. In glaucoma—the world’s most prevalent optic neuropathy [52]—degeneration proceeds from stress asserted at the optic nerve head in both directions. A distal program includes early degradation of anterograde axon transport from retina to central brain targets, followed by disassembly of the myelinated axon as part of frank degeneration of the optic nerve [24]. Importantly, RGC bodies and the unmyelinated axon segment in the retina persist until late in progression [22]. This persistence lies at the root of hope for regenerative therapies to restore the distal axon projection and visual signaling to the brain [40,41]. Similarly, RGC loss following acute trauma to the eye or anterior skull involves early axon dysfunction with subsequent axon and somatic degeneration [47,53,54]. Both glaucoma and optic nerve trauma involve secondary inflammation that appears to exacerbate progression [41,47,55]. This also applies for models of trauma utilizing optic nerve crush [56,57], which is the most prevalent experimental system for studying RGC axon repair and regeneration.

Recently, we found that a collagen mimetic peptide (CMP 03A) prevented degradation of RGC axonal transport to the superior colliculus (SC), the primary target for RGC axons in rodents [24,25], following three weeks of microbead-induced elevations in intraocular pressure (IOP) [19]. This same CMP promoted healing of the corneal epithelium following acute corneal surface injury [58]. Additional members of the CMP family were efficacious in promoting neurite outgrowth from dorsal root ganglia cultures challenged by matrix metalloproteinase (MMP) digestion of collagen plating [19]. Here, we found that intravitreal delivery of one of these (CMP 13A) prevented 67% of degradation in anterograde transport following three weeks of IOP elevation (Figure 2), bringing transport levels to those of the vehicle saline eye (i.e., normal IOP; *p* = 0.06). This rescue had the effect of restoring the retinotopic representation of functional axonal transport in the SC (Figure 2, top). This is an important finding, given that elevated IOP is a prevalent risk factor for optic nerve degeneration in glaucoma [30]. By restoring transport to levels in the control eye for the vehicle group, the protective influence of CMP 13A is similar in magnitude to that of other experimental strategies we have tested that also protect transport and prevent frank degeneration [26,27,48,49]. Interestingly, we also found that transport from the control eye in the CMP 13A cohort significantly exceeded that from the corresponding eye in the vehicle cohort (Figure 2). We speculate that this could be due to CMP-mediated repair of minor damage to the collagen matrix in the tissues contralateral to IOP elevation due to low-grade systemic inflammation or transfer of other pathogenic signals between the two eyes [43]. We found a similar trend in our previous study using CMP 03A, though the increase in transport in the CMP-treated saline eye did not reach significance [19]. 

We also extended our studies of the reparative properties of CMPs to an acute model of optic neuropathy: optic nerve crush. Intravitreal delivery of CMP 13A three days following the crush accelerated the repair of RGC axons, as shown by the greater abundance of CTB-containing axons in the optic nerve on the distal side (towards the brain) of the injury site compared to nerves from the vehicle group (Figure 3). The presence of CTB in an axon is indicative of its successful functional transport from the RGC body in the retina, a process that requires intact active uptake and transport [25]. Due to tortuosity in individual axons, it is difficult to visualize an entire axon in a given section of nerve. However, CMP 13A nerves demonstrated a greater abundance of axon segments containing CTB at further distal sites along the nerve (Figure 4A,B). The segments that were visualized were also longer on average, with CMP-treated nerves demonstrating the longest intact segments in the cohort (Figure 4C). This indicates a higher degree of intact RGC axon transport along the nerve, and thus a greater degree of repair due to the CMP. Interestingly, we often observed that CTB-containing axon segments co-localized with pockets of CMP 13A, visualized through its attached fluorescent moiety (Figure 3B). While this was not a significant correlation (*p* = 0.18), it does raise the possibility that intravitreal injection allows for sufficient bioavailability of CMP to bind to disrupted collagen at even more distal sites outside of the eye. Alternatively, one could speculate that CMP binds to damaged collagen in the extra-axonal matrix that migrates along with recovering axon segments. Both possibilities leverage the well-established property of the CMP sequences we utilize to selectively intercalate into damaged collagen strands [14]. 

Our results do not preclude the possibility of CMPs directly repairing RGC axonal membranes, though collagen is nearly exclusively localized in the extracellular domain within the matrix. That CMP delivery both rescues intra-axonal transport of CTB taken up by RGCs (Figure 2) and repairs axons damaged by crush (Figure 3 and Figure 4) hints at a common, likely extracellular mechanism of action. In the optic nerve projection, both chronic disease and acute injury elicit a cycle of degradation and remodeling of the ECM, including collagen content [5,7,34,36,37,59,60]. This process involves important interplay between matrix metalloproteinases and tissue inhibitors of MMPs [61], the balance of which indicates reparative remodeling, especially following trauma [62]. Repairing damaged collagen could be an important means to restore function quickly, by tamping down pathogenic processes. As matrix collagen degrades in the CNS, formation of a glial scar by reactive astrocytes secreting an overabundance of collagen type IV creates a barrier to axon repair and regeneration by binding inhibitory molecules and inducing migration of inflammatory microglia and other immune cells [63,64,65,66]. Thus, repairing matrix fragments early in disease or injury could restore axon function before the onset of subsequent stages of degeneration that necessitate the more difficult barrier of regeneration over restoration.

## 4. Materials and Methods 

### 4.1. Inducible Glaucoma Model: Microbead Occlusion 

Recently, we found that multiple CMPs accelerated neurite outgrowth from dorsal root ganglia plated on collagen challenged by digestion with matrix metalloproteinase-1 (MMP-1) [19]. Here, we tested whether one of these (CMP 13A) is efficacious in protecting the RGC projection under conditions of elevated IOP induced by microbead occlusion. This CMP was manufactured in limited quantities by Bachem, AG (Germany), with the Tide Fluor™ 2 moiety (AAT Bioquest, Sunnyvale, CA, USA) as a fluorescent reporter and has sequence (cis-Flp-Hyp-Gly)_7_, where hydroxyproline (Hyp), glycine (Gly), and 4-fluoro-proline (Flp) are abbreviated as indicated. This CMP is analogous to the 7-repeat, 21-residue sequences previously shown to not spontaneously form triple helices as they intercalate with high affinity and selectivity into damaged type I collagen in vitro and in vivo [13,14,15]. 

The Vanderbilt University Institutional Animal Care and Use Committee approved all experimental procedures. We used microbead occlusion to elevate intraocular pressure (IOP) unilaterally in 6- to 8-week-old male C57BL/6 mice (n = 7 per cohort; Charles River Laboratory, Wilmington, MA), following our published protocol [20,21,43,44,45,67,68]. We obtained bilateral IOP measurements 2–3 days prior to elevation in anesthetized (2.5% isoflurane) mice using TonoPen XL (Medtronic Solan, Minneapolis, MN, USA) and averaged these to obtain a baseline measure (day 0). We elevated IOP unilaterally by injecting 1.5 µL of 15 µm polystyrene microbeads (approximately 1500 microbeads; Invitrogen, Carlsbad, CA, USA) into the anterior chamber using a borosilicate glass pipette trimmed to a diameter of 100 µm and attached to a micromanipulator; the fellow eye received an equivalent-volume saline injection as a control. Both eyes received vehicle (phosphate-buffered saline, PBS) or CMP 13A (in PBS, 100 µM) via a single intravitreal injection (1.5 µL) at day 10 following microbead/saline injections. We measured IOP regularly during the three-week experimental period while mice were maintained on a 12 h light–dark cycle with standard rodent chow available ad libitum. 

### 4.2. Acute Injury Model: Optic Nerve Crush

Retrobulbar optic nerve crush (ONC) is a useful model of traumatic optic neuropathy and for assessing reparative and regenerative strategies for RGC axons [50]. In additional C57BL/6 mouse cohorts, we performed bilateral optic nerve crush intra-orbitally following intraperitoneal injection of ketamine. Following our established protocol [69], we visualized the optic nerve without disrupting the orbital sinus using a surgical microscope and used self-closing forceps to apply crush for 10 s, about 1 mm distal to the central artery entrance to the globe. Both eyes received a single intravitreal injection (1.5 µL) of either CMP 13A (200 µM) or vehicle (DMSO) three days following crush. 

### 4.3. Cholera Toxin B Assessment of RGC Axon Transport in Nerve and Brain

Three weeks following microbead injection and twelve days following nerve crush, we anesthetized mice with 2.5% isoflurane and injected both eyes intravitreally with 1.5 μL of 1 mg/mL solution of cholera toxin subunit B (CTB) conjugated to Alexa-555 (Molecular Probes, Eugene, OR, USA) following our published protocol [20,43,45,46]. Two days later, the mice were perfused transcardially with PBS followed by 4% paraformaldehyde, and dissected tissues were cryoprotected in 30% sucrose. For mice in the experimental glaucoma cohorts, we prepared coronal midbrain sections (50 µm thick) on a freezing sliding microtome and photographed sections of the superior colliculus using a Nikon Ti Eclipse microscope (Nikon Instruments Inc., Melville, NY, USA). As described in our recent publications [19,43,68,70], we quantified the intensity of the CTB signal (intact transport) within the collicular retinotopic map using a custom ImagePro macro (Media Cybernetics, Bethesda, MD, USA). 

For mice in the crush cohorts, we prepared 10 µm-thick longitudinal sections through the optic nerves and obtained micrographs of CTB-labeled RGC axons using an Olympus FV1000 confocal microscope with a 40× oil immersed objective. The injury site was identified independent of the CTB label by a combination of (1) narrowing of the nerve, (2) obvious disruption of tissue or cell abundance based on immune labeling, and (3) higher level of autofluorescence in an unused color channel. At discrete distances extending distally from the lesion site, we used ImageJ to measure the width of the nerve and scored the number of CTB+ RGC axon segments crossing the line joining the nerve edges. The number of axons per width was then scaled to the average width of the optic nerve (250 μm). We measured the length of contiguous axon segments extending beyond the lesion site using the semi-automated tracing Simple Neurite Tracer (SNT) plugin within the Fiji distribution of ImageJ [71]. This tool traces a path with user guidance along the axon segment and then calculates a total length as the sum of vectors. In neither microbead nor crush models did we observe gross ocular inflammation, irritation, or other pathology associated with intraocular injections or treatment.

### 4.4. Statistical Analysis

All data are presented as mean ± standard error of the mean (SEM). Statistical analyses and graphs were made using Sigma Plot Version 14 (Systat, San Jose, CA, USA). Outlier analysis was performed using Grubbs’ test (Graphpad Software, San Diego, CA, USA). Parametric statistics were performed (*t*-test, analysis of variance) if data passed normality and equal variance tests; otherwise, we performed non-parametric statistics (Mann–Whitney, ANOVA on ranks, Welch’s test). Statistical significance was defined as *p* ≤ 0.05. 

## Data Availability

The data presented in this study are available in Figure 1, Figure 2 and Figure 4.
